# Within-pair analysis of monozygotic twins after bariatric surgery: a systematic review

**DOI:** 10.1007/s11695-026-08730-1

**Published:** 2026-06-02

**Authors:** Benjamin Alfonso Thorpe-Plaza, Javier Baltar, Fernando Santos, Purificación Parada-González

**Affiliations:** 1 University of Santiago de Compostela, Rua Choupana sin número, Santiago de Compostela, Spain; 2 Servicio Gallego de Salud, Rua Choupana sin número, Santiago de Compostela, Spain

**Keywords:** Surgicogenomics, Metabolic and bariatric surgery, Monozygotic twins

## Abstract

Obesity is an increasingly widespread illness currently affecting more than 890 million adults worldwide. Excess body weight is a multifactorial challenge, driven by complex interaction between genetic predisposition and environmental influences rendering the underlying causes of obesity difficult to identify. Monozygotic twin (MZT) studies provide great value in medical research analyzing external exposures within twin pairs with fixed genetic or environmental factors that normally tend to confound results in general population studies. Metabolic-bariatric surgery (MBS) is considered an effective durable therapy in terms of body weight loss (BWL) and the control of cardiometabolic risk factors in obese patients. Body mass index (BMI) is known to be strongly related in MZ twins but whether this relation is stable between MZ twins after MBS is still to be determined. The results of this study support the importance of genetics over BWL after MBS with high correlation amongst MZ twin pairs.

## Introduction

Obesity is considered a chronic, metabolic disease responsible for multiple comorbidities including type 2 diabetes, hypertension, dyslipidemia, cardiovascular diseases, non-alcoholic fatty liver disease, Alzheimer, as well as cancer in response to the chronic inflammatory state to which obese patients are overexposed [1–6]. Metabolic-bariatric surgery (MBS) has been reported as the most successful clinical intervention in severe obesity, achieving a doble effect by consistent rapid, sustainable body weight loss (BWL) as well as a restoration of the metabolic homeostasis [3, 4]. However, not all patients are feasible for surgery and therefore each case should be assessed individually by a multidisciplinary team of experts addressing all therapeutic options available, balancing from healthy lifestyle habits to psychological evaluation, pharmacotherapy and when necessary, invasive endoscopic procedures or surgical interventions [1, 4, 6–8].

There are multiple factors affecting obesity and although genetics have proven to be a determinant role in BWL after MBS, little is known regarding surgicogenomics [9, 10]. Steps have been made attributing higher BWL after MBS to recently identified gene mutations likewise MC4R gene mutation [11, 12], ST8SIA2 omental fat expression [10] or non-A allele carriers of rs9939609 genetic variant of the FTO gene which showed higher BWL and reduction of type 2 diabetes after MBS [13]. Even though the role of genetics is firm [9–11, 14, 15], it doesn´t account for the complete obese phenotype [6]. Epigenetic printing patterns have been identified in response to weight loss in MZT pairs followed by either hypocaloric diets or MBS. Although MBS shows a greater impact on methylation profile when compared with hypocaloric diets, both weight-loss methods can restore obese patients DNA-methylation patterns that resemble those in healthy population [3, 6, 8]. MBS epigenetic modifications seem to have a crosslink effect over DNA methylome responsible for metainflamation, modulating and improving not only obesity but also obesogenic related comorbidities [3, 4].

The complex intertwined interactions between genetics and environmental factors render the underlying causes of obesity difficult to elucidate [4, 5, 16], making MZT study models a valuable opportunity to analyze the comorbidities behavior and BWL outcomes after MBS in patients with fixed genetic background [16]. Multiple case reports have been published regarding single MZ twins operated of MBS, however, further conjoint analysis of results are needed to achieve further conclusions. The purpose of this systematic review is to present all MZ twin pairs operated of MBS to the present date, comparing and analysing the repercussion of MBS over BWL under the same genetic context.

## Methods

### Literature search

**Fig. 1:**
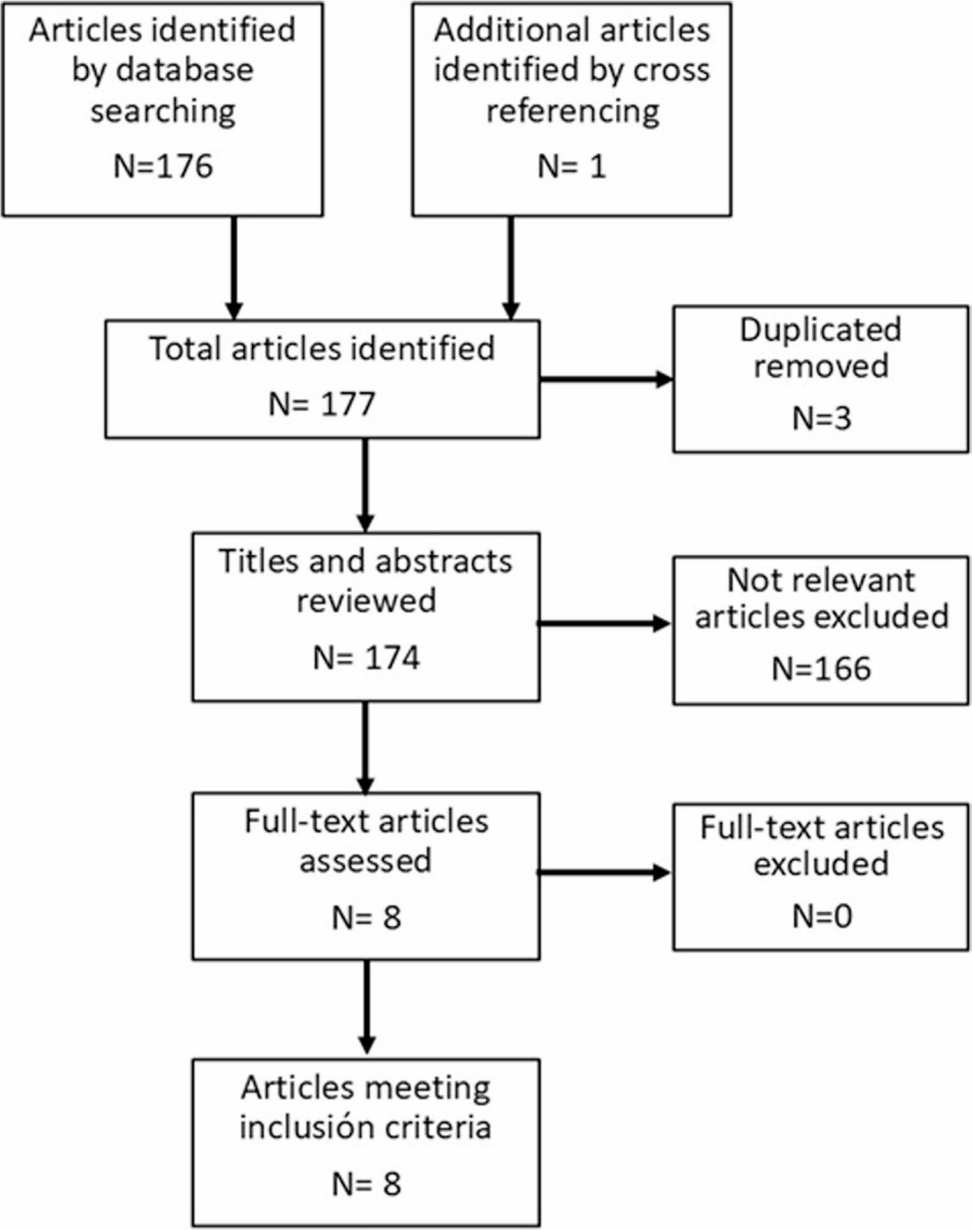
Prisma flow diagram of the article selection process

This study was constructed based on the Preferred Reporting Items for Systematic Reviews and Meta-Analyses (PRISMA) guidelines (24). Online research was performed including all the articles published between September 1999 and August 2025 assessing the topic of monozygoitic twin patients submitted to bariatric surgery. The searching strategy included the following keywords: ¨bariatric surgery¨, ¨metabolic surgery, ¨obesity surgery¨ and ¨twins¨. The databases included on this search were PubMed, Medline, CINAHL and Cochrane library. Duplicated articles were deleted. 8 articles were finally selected for this systematic review, out of which 2 were case series and 6 were twin pair case reports. Contact with one of the authors (2) was made with no answer on their behalf, no other contact with article´s authors were made Fig. 1.

### Article exclusion criteria

Regarding the little data of twins operated on bariatric surgery and with the aim of studying their intra pair weight differences during the postoperative follow up, there were no exclusion criteria on the found case series and case reports.

### Data collection process

Data collection was performed independently in each case report. Firstly, the data concerning demographic and surgical characteristics were collected and summarized on the following parameters: Age, sex, presurgical endoscopy, Type of surgery, same surgical team, immediate postoperative complications, hospital stay, time gap between surgeries and time of follow up. A subsequent descriptive statistical analysis was performed. Secondly, the data regarding weight evolution was collected, the information regarding the patient´s weight was inconsistent, with some cases presenting the patient´s weight in Kgs and other articles using body mass index (BMI). Three twin pairs lacked the data concerning their weight evolution and therefore were not included in the ensuing statistical analysis. The rest of patient´s weight data were standardized and summarized through preoperative BMI and BMI control which permitted to calculate twin´s total weight loss (TWL %) and intrapair TWL differences and Estimated body mass index loss (EBMIL %) and intrapair EBMIL differences. Pearson´s correlation coefficient and intraclass correlation coefficient (ICC) were calculated for TWL and EBMIL. Information regarding glycaemic and lipid preoperative and postoperative levels were collected but insufficient to present analytic statistical analysis.

## Results

8 articles were identified describing 12 cases of MZT pairs surgically treated for obesity Table 1:

**Table 1:** Demographic and surgical characteristics

Case report	Sex/Age at surgery	presurgical Endoscopy	Type of Surgery	Same surgical team	PO complications	Hospital Stay	Time between surgeries	Follow up
Fried/1999	Female/-		Laparotomic nonadjustable gastric band/Laparoscopic nonadjustable gastric band					12 m
	Female/-		Laparoscopic Nonadjustable gastric band					12 m
Hagedorn/2007	Female/49		Laparotomic Gastric bypass	Yes	No			24 m
	Female/29		Laparoscopic Gastric bypass	Yes	No		Same Year	12 m
	Female/29		Laparoscopic adjustable gastric band	No	No		Same Year	12 m
	Female/38		Laparoscopic gastric bypass	Yes	No		Same day	12 m
Parmar/2022	Female/28	Yes: Twin A 2 cm hiatus hernia (HH) Twin B 3 cm HH.	Laparoscopic one anastomosis gastric bypass	Yes	No	48 h	2 weeks	24 m
Nicholas/2022	Male/30	No	Laparoscopic gastric bypass/laparoscopic gastric bypass and Ladd’s procedure.	Yes	No		5 months	12 m
Guimaraes/2023	Female/30		Laparoscopic Biliopancreatic diversion with duodenal Switch/SADI-s	Yes	No		Same day	12 m
Huseynov/2023	Female/37	Yes: both Normal	Laparoscopic Sleeve gastrectomy	Yes	No	48 h	Few days	18 m
Menon/2024	Female/49	Yes: both normal. Gastric balloon placement.	Laparoscopic SADI-s	Yes	No	48 h	Few weeks	36 m
Thorpe/2024	Female/29 and 31	Yes: both Normal	Laparoscopic Gastric bypass	Yes	No	72 h	2 years	36 m and 60 m.

**Fig. 2:**
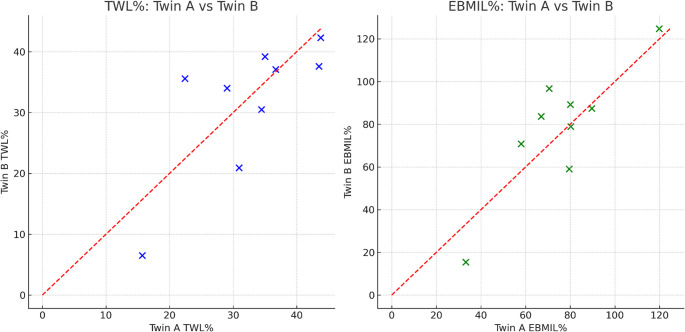
Scatter plots showing the correlation of weight loss outcomes (TWL% and EBMIL%) between Twin A and Twin B

**Fig. 3:**
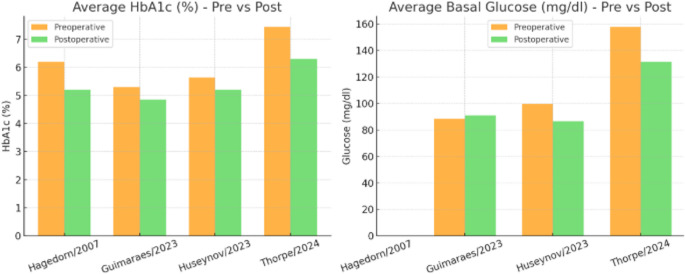
Comparison of preoperative and postoperative average HbA1c levels (%) and basal glucose (mg/dL) across the selected studies

Analyzing the current bibliography, we observe the following demographic data: 24 patients distributed in 12 pairs of MZT surgically treated for obesity. Regarding their sex, 11/12 pairs (91,66%) were female and 1/12 pair (8,33%) were male. The age range varied from 29 to 49 years with a median age of 30 years (Figs. 2 and 3).

From the total sample 4/12 pairs (33,33%) described preoperative endoscopy in their case reports. One pair of twins (3) presented pathological endoscopies describing small hiatal hernias of 2–3 cm in both twins. Another pair of twins (4) had placed a gastric balloon, prior to the bariatric surgery, as bridging therapy for definitive bariatric surgery.

Out of the entire sample, 11/12 pairs (91.66%) received the same surgical procedure within siblings, and 1/12 pairs (8.33%) received different surgical procedures. The most common procedure was the gastric bypass 5/12 (42%) followed by the gastric band 3/12 (25%) (2 nonadjustable, 1 adjustable). One pair of twins underwent one anastomosis gastric bypass 1/12 (8,33%), another twin pair underwent sleeve gastrectomy 1/12 (8,33%), and another pair SADI-s 1/12 (8,33%). The remaining twin pair received different surgical procedures each, one a Biliopancreatic diversion with a duodenal switch and the other one a SADI-s 1/12 (8,33%).

**Fig. 4:**
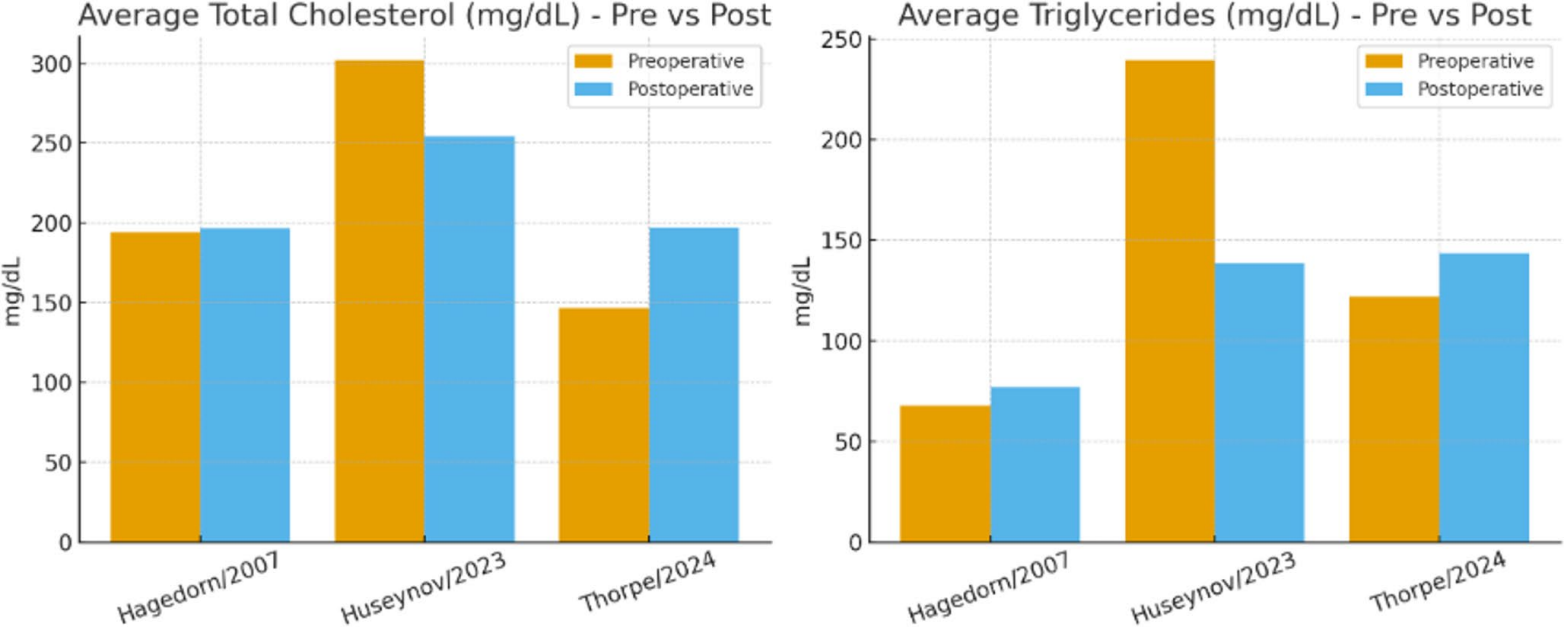
Comparison of preoperative and postoperative average total cholesteroland triglyceride levels across different|studies

Except for one case (nonadjustable gastric band) all the patients were operated on by the same surgeon/surgical team. The information given regarding the gap of time between surgeries is not collected in detail, however with the given information we can state that the time gap between surgeries amongst twin pairs varied from the same day to 2 years. In 11/12 pairs (91.66%) both surgeries were performed in the same year. Regarding the hospital stay, only 4/12 (33,33%) studies collected this data, three presented a 48-hour hospital stay and one presented a 72-hour hospital stay. No immediate postoperative complications were described in any of the case reports Fig. 4.

The postoperative follow-up was collected in all the case reports, with a minimum follow-up of a year. Most twin pairs were followed in outpatient consultation for 12 months 7/12 (58,33%), one twin pair was followed for 24 months 1/12 (8,33%), another twin pair for 18 months 1/12 (8,33%) and the two resting twin pairs for a minimum of 36 months 2/12 (16,66%) Table 2.

**Table 2:** Weight evolution

Case report	BMI Initial	Time of follow up (months)	BMI control	TWL (%)	Intrapair TWL difference (%)	EBMIL (%)	Intrapair EBMIL difference (%)
Fried/1999		12					
		12					
Hagedorn/2007	Twin A: 54.9 Twin B: 53.5	24	Twin A: 30.9 Twin B: 30.9	Twin A: 43.77 Twin B: 42.3	1.47	Twin A: 80.27 Twin B: 78.95	1.32
	Twin A: 45.1 Twin B: 42.7	12	Twin A: 29 Twin B: 26.9	Twin A: 36.7 Twin B: 37.09	0.39	Twin A: 80.01 Twin B: 89.27	9.26
	Twin A: 47.3 Twin B: 43.4	12	Twin A: 39.9 Twin B: 40.6	Twin A: 15.7 Twin B: 6.5	9.20	Twin A: 33.18 Twin B: 15.43	17.75
	Twin A: 52.4 Twin B: 47	12	Twin A: 34.1 Twin B: 28.6	Twin A: 35 Twin B: 39.2	4.20	Twin A: 66.94 Twin B: 83.64	16.7
Parmar/2022	Twin A: 48.4 Twin B: 45.1	12	Twin A: 27.43 Twin B: 27.53	Twin A: 43.5 Twin B: 37.6	5.9	Twin A: 89.62 Twin B: 87.41	2.21
Nicholas/2022	Twin A: 41.6 Twin B: 36.7	12					
Guimaraes/2023	Twin A: 50.04 Twin B: 48.11	12	Twin A: 35.51 Twin B: 31.74	Twin A: 29.03 Twin B: 34.03	5.00	Twin A: 58.02 Twin B: 70.84	12.82
Huseynov/2023	Twin A: 40.3 Twin B: 37.1	12	Twin A: 31.13 Twin B: 25.4	Twin A: 22.43 Twin B: 35.6	13.17	Twin A: 70.5 Twin B: 96.7	26.2
Menon/2024	Twin A: 35.1 Twin B: 33.1	36	Twin A: 23 Twin B: 23	Twin A: 34.47 Twin B: 30.5	3.97	Twin A: 119.8 Twin B:124.69	4.89
Thorpe/2024	Twin A: 40.6 Twin B: 38.2	12	Twin A: 28.2 Twin B: 30.4	Twin A: 30.92 Twin B: 20.93	9.99	Twin A: 79.49 Twin B: 59.09	20.4

Even though the 12 reported cases presented a minimum follow-up of 12months, only 9/12 reflected a registry of the weight evolution. Out of these studies, with the given information in each case report, it was possible to calculate the BMI prior to the surgery (Initial BMI), the postoperative BMI (BMI control), the TWL%, the EBMIL% and the intrapair differences of TWL and EBMIL.

The average value of intrapair TWL% difference was 5.92% with a Standard deviation of 4.15% and a confidence interval (2.73%−9.11%).

Pearson correlation coefficient TWL%: r = 0.749 (p = 0.020).

ICC TWL%: ICC ≈ 0.949.

The average value of intrapair EBMIL% difference is 12.39% with a standard deviation of 8.62% and a confidence interval (5.77%−19.02%).

Pearson correlation coefficient EBMIL%: *r* = 0.860 (*p* = 0.003).

ICC EBMIL%: ICC ≈ 0.917.

Table 3 should appear above the graphic containing information of Table 3.

**Table 3:** Pre and postoperative glycemic values

Case report	Preoperative HbA1c (%)	Preoperative basal glucose values (mg/dl)	Postoperative HbA1c (%)	Postoperative basal glucose values (mg/dl)
Hagedorn/2007	Twin A: 5.8 Twin B: 6.6		Twin A: 5.1 Twin B: 5.3	
Guimaraes/2023	Twin A: 5.3 Twin B: 5.3	Twin A: 83 Twin B: 94	Twin A: 4.7 Twin B: 5	Twin A: 90 Twin B: 92
Huseynov/2023	Twin A: 5.68 Twin B: 5.6	Twin A: 96 Twin B: 103	Twin A: 5.2 Twin B: 5.2	Twin A: 82 Twin B: 91
Thorpe/2024	Twin A: 6.6 Twin B: 8.3	Twin A: 109 Twin B: 207	Twin A: 6 Twin B: 6.6	Twin A: 114 Twin B: 149

Out of the entire sample 4/12 pairs (33,33%) monitored the glycemic preoperative and postoperative values, showing in all cases a mild decrease with better overall glycemic controls. In our previous case report, the patients presented similar glucose values but the need for antidiabetic drugs was reduced from insulin to oral treatment.

Table 4 should appear above the graphic containing information of table 4.

**Table 4:** Pre and postoperative lipid values

Case report	Preoperative total cholesterol (mg/dl)	Preoperative triglycerides (mg/dl)	Postoperative total cholesterol (mg/dl)	Postoperative Triglycerides (mg/dl)
Hagedorn/2007	Twin A: 196 Twin B: 192	Twin A: 72 Twin B: 64	Twin A: 188 Twin B: 205	Twin A: 60 Twin B: 94
Huseynov/2023	Twin A: 278 Twin B: 326	Twin A: 209 Twin B: 270	Twin A: 220 Twin B: 289	Twin A: 128 Twin B: 149
Thorpe/2024	Twin A: 137 Twin B: 156	Twin A: 106 Twin B: 138	Twin A: 186 Twin B: 208	Twin A: 123 Twin B: 164

Only 3/12 twin pairs (25%) registered the values of preoperative and postoperative cholesterol and triglycerides showing mixed results. Postoperative total cholesterol values could be deceived due to the increased levels of HDL. A greater sample is required to correctly define a clearer overall clinical tendency.

## Discussion

The results of this systematic review including the available data of MZT surgically treated for obesity, shows a strong correlation between BWL after MBS amongst identical siblings, despite differences in type of surgery, environmental influences, age, sex or time interval between surgeries. This supports the results of previous studies by Hatoum et al. which highlighted the importance of genetics on BWL after RYGB not only as a response to dietary restriction but mainly due to metabolic-hormonal physiological postoperative modifications [10, 15]. The analysis of the collected data of MZT includes diverse types of surgical procedures, from RYGB, one anastomosis gastric bypass, SADI-s, gastric bands, sleeve gastrectomy to biliopancreatic diversion with duodenal switch (BPD/DS) [1, 16–22], suggesting that the role of genetics over postoperative BWL is not only patent after RYGB but also after many other MBS procedures.

Shared genetics confers on MZT studies up to seven times higher statistical power than traditional randomized controlled trials [23] favoring the growth of twin registries worldwide with MZT research databases [24–28] aiming to uncover environmental sensitives and specific genetic predispositions for obesity and other pathologies under controlled genetic variability [29]. Multiple studies over the past years have worked on identifying potential factors responsible for higher rates of postoperative BWL, as their identification, could enable profiling patients in whom MBS would be more suitable [10, 15, 16]. Despite the already described greater correlations between MZT and BWL after low calory diets [3] or the higher BMI correlation amongst MZ twins who present bad eating habits regardless their different environmental backgrounds, the specific biological mechanisms responsible of BWL after MBS remain unclear [29].

Hatoum et al. compared %BWL followed by MBS amongst first-degree relatives with different households to non-relatives which cohabitated together, results showed a similar BWL amongst first-degree relatives with 9% EBMIL mean difference, which was not observed within non-relatives who cohabitated together [9, 14, 15]. Stunkard et al. described how despite reared apart, twin´s BMI intrapair correlation remains similar despite different environmental exposure [18, 29]. Such results have led to attribute great importance to genetics, suggesting that up to 70% of variability in BWL after MBS is deemed to genetic predisposition [10, 15, 18]. Same tendency results are evident on this systematic with a similar BWL tendency in terms of TWL% and EBMIL% with CCI values > 0.80 despite undergoing different types of surgical procedures. This data supports the hypothesis that genetic factors substantially influence BWL after MBS. However, although most MZ twin pairs showed moderate intrapair differences, a subset exhibited discordances above p75 of the distribution suggesting that, even among genetically identical individuals, non-genetic factors such as the clinical timing of surgery, the surgical team, and the perioperative care environment may significantly influence weight-loss outcomes after MBS [19]. The impact of these factors may be more pronounced in the early postoperative follow-up and could attenuate over time, potentially leading to a progressive intrapair convergence of BWL; however, this hypothesis warrants confirmation through longer-term follow-up.

When analysing the comorbidities behaviour in MZ twins after MBS little data is available in the current bibliography. Hormonal profile and metabolic outcomes vary up on different MBS interventions as their mechanism of action are different [20, 30]. Understanding the intertwined responsibility of environmental and genetic factors tends to dissipate when studying identical siblings as shown by Guimarães et al., where a single pair of MZ twins underwent different MBS procedures, a SADI-s and a BPD/DS. Results showed diverged hormonal and metabolite postoperative patterns, with a more conservative tendency in SADI-s suggesting a different weight loss mechanism, unlike it was previously suggested [20] Similar results were evident, in our experience, after several revision surgeries from BPD/DS to SADI-s, preserving the metabolic and bariatric effect after SADI-s but milder than BPD/Ds [30]. Either in within-subject studies or within – twin pairs studies, genetic variability remains constant, helping conclude that longer common channel in SADI-s is responsible for different metabolic outcome, when compared with BPD/DS, as well as for lower rates of malnutrition [20, 30].

Cholesterol and triglycerides levels improve in at least 70% of patients undergoing MBS in non-genetically related population [8]. Specifically, in MZ twins, a similar tendency is shown with overall improvement regarding cholesterol [1, 10, 19, 22], with a parallel behaviour on LDL levels within MZ twin pairs [1, 19, 21, 22] as well as cardiometabolic protective range levels of HDL [19]. A similar trend is evidenced regarding the postoperative glucose metabolism, with a generalized amelioration of diabetes control [1, 20, 22] with cases of insulin withdrawal leading to reported increased patient´s quality of life and resolution of insulin resistance [19]. Despite the scarce data available in the published case reports regarding glycaemic profile tendency, there is a common mild decrease in the glucose and haemoglobin glucoside (HbA1c) levels after MBS [1, 19, 20, 22].

This paper has several limitations due to the small cohort sample available in the current literature regarding MZ twins operated of MBS and more so the short available information regarding pre and postoperative clinical aspects of obese patients in terms of comorbidities and environmental factors. Despite achieving interesting conclusions regarding postoperative BWL, the available information regarding cholesterol and glycaemic behaviour were insufficient to describe overall relevant clinical tendencies.

## Data Availability

No datasets were generated or analysed during the current study.
